# Antimicrobial Resistance and Therapy in the Intensive Care Unit

**DOI:** 10.3390/antibiotics14090904

**Published:** 2025-09-08

**Authors:** Pablo Vidal-Cortés, Borja Suberviola-Cañas, David Andaluz-Ojeda

**Affiliations:** 1 Intensive Care Department, Complejo Hospitalario Universitario de Ourense, 32003 Ourense, Spain; pablo.vidal.cortes@sergas.es; 2 Intensive Care Department, Hospital Universitario Marqués de Valdecilla, 39008 Santander, Spain; borja.suberviola@scsalud.es; 3 Intensive Care Department, Complejo Universitario Asistencial de Palencia, 34005 Palencia, Spain

Antimicrobial resistance (AMR) has emerged as one of the greatest public health challenges in the last decades. Despite considerable investment and scientific effort, bacteria continue to develop new resistance mechanisms soon after the introduction of novel antibiotics. Critically ill patients are particularly vulnerable to infections caused by multidrug-resistant (MDR) microorganisms due to their severe underlying conditions, frequent exposure to invasive procedures, and high utilization of broad-spectrum antimicrobials. When infection occurs in such patients, timely and appropriate treatment becomes a race against time, with therapeutic success or failure often determined within the first hours of clinical management.

In this context, clinicians are confronted with the need to be familiar with local epidemiology, novel microbiological diagnostic tools, and the pharmacological properties of both traditional agents and newer antibiotics. The current antimicrobial arsenal is more diverse than ever before, with agents differing not only in their spectrum and mechanisms of action, but also in their safety profiles and pharmacokinetic/pharmacodynamic (PK/PD) properties, which can significantly influence outcomes.

This Special Issue of Antibiotics brings together ten original studies and reviews that comprehensively address the epidemiology, therapeutic strategies, and optimization of antimicrobial use against key MDR pathogens in ICU settings.

The issue opens with the large epidemiologic multicentre study by Kilinc et al. [1], which examines the burden of AMR in critically ill patients and its profound impact on mortality. Their findings underscore the critical importance of rapid, targeted therapy, particularly in infections caused by *Acinetobacter baumannii* and *Pseudomonas aeruginosa*, two pathogens notorious for their resistance profiles and poor clinical outcomes.

This provides a natural segue into the work of Rodríguez-Aguirregabiria et al. [2], who evaluate cefiderocol as a therapeutic option against carbapenem-resistant *A. baumannii* (CRAB) infections in ICU patients. Their results support the role of cefiderocol as a valuable alternative, while also identifying concerning resistance in approximately 20% of isolates, notably those harbouring blaNDM-1 carbapenemase and mutations in the AmpC gene. The discussion on difficult-to-treat pathogens continues with the review by Vidal-Cortés et al. [3] on *P. aeruginosa.* They present a comprehensive review covering epidemiology, resistance mechanisms, risk factors as well as future therapeutic options. In addition, the authors explore strategies for optimizing treatment, including the integration of PK/PD principles and combination therapy approaches to improve outcomes in this challenging clinical context.

Attention then shifts to *Stenotrophomonas maltophilia*, an emergent significant MDR pathogen in ICUs. One review and a research article address the treatment of this increasingly prevalent resistant microorganism in many ICUs. Carbonell et al. [4] provide valuable insights into its management, discussing antimicrobial susceptibility patterns and the importance of tailored therapeutic strategies. Complementing this, Barrasa et al. [5] apply Monte Carlo simulations to evaluate PK/PD target attainment in the optimization of antibiotic therapy for *S. maltophilia* infections in critically ill patients, offering a practical framework for dose adjustment in complex scenarios.

Another impactful contribution in this Special Issue is the meta-analysis by Risco-Risco et al. [6], which assesses the efficacy of cefiderocol for severe MDR Gram-negative infections in ICU patients. Their pooled analysis suggests adequate safety profile and significant benefits, including improved survival especially in CRAB infections, reinforcing cefiderocol’s role in the therapeutic arsenal against these challenging infections.

Beyond the choice of the right drug, administration strategy is a crucial determinant of clinical success. Continuous and extended infusions of β-lactams have gained prominence, with emerging evidence linking these approaches to improved clinical cure and even survival rates in severe infections. In this context, Hyun et al. [7] compare extended versus intermittent meropenem infusion in ICU patients with severe MDR infections, finding a reduction in mortality with extended infusion, supporting the broader adoption of optimised dosing strategies. In this sense, Therapeutic Drug Monitoring (TDM) is further explored by Gatti et al. [8], who investigate meropenem administration in continuous infusion in critically ill patients receiving continuous renal replacement therapy (CRRT). Their findings are particularly noteworthy: more than 80% of patients required dose adjustment after the first drug level measurement, and with this innovative technique for optimizing antibiotic treatment over 90% ultimately survived—figures exceeding expected survival rates in such critically ill populations.

Finally, looking beyond systemic antimicrobial therapy, innovative therapeutic approaches in severe infections treatment are presented in this issue. Ruiz-Rodríguez et al. [9] describe individualized, phenotype-driven management in a case series of streptococcal toxic shock syndrome using adjuvant therapies as hemoadsorptive techniques or immunoglobulins infusion in selected patients, while Hajska et al. [10] evaluate the antimicrobial efficacy of impregnated human acellular dermal substitutes in burn wound models. These pioneering studies open new avenues for adjunctive or alternative therapies, aiming to improve outcomes in the most complex and fragile ICU patients.

Collectively, the contributions in this Special Issue provide a comprehensive and clinically relevant overview of the multifaceted challenge posed by MDR pathogens in ICUs. From large-scale epidemiological analyses to detailed PK/PD optimisation studies and novel therapeutic strategies, these works highlight both the progress made and the hurdles that remain. The evidence presented here will undoubtedly serve as a valuable reference for intensivists, infectious disease specialists, and clinical microbiologists striving to improve outcomes in this high-risk patient population.
